# Rutin attenuates Tramadol-induced lung injury in rats by modulating oxidative stress, inflammation, endoplasmic reticulum stress, and apoptosis

**DOI:** 10.1007/s00210-026-05314-9

**Published:** 2026-04-18

**Authors:** Mustafa Önder Gönen, Nurhan Akaras, Hasan Şimşek, Özge Kandemir, Cuneyt Caglayan, Hüseyin Mutlu, Fatih Mehmet Kandemir

**Affiliations:** 1 Department of Emergency Medicine, Meram State Hospital, Konya, Turkey; 2 Department of Histology and Embryology, Faculty of Medicine, Aksaray University, Aksaray, Turkey; 3 Department of Physiology, Faculty of Medicine, Aksaray University, Aksaray, Turkey; 4 Department of Food Processing, Aksaray Technical Sciences Vocational School, Aksaray University, Aksaray, Turkey; 5 Department of Medical Biochemistry, Faculty of Medicine, Bilecik Seyh Edebali University, Bilecik, Turkey; 6 Department of Emergency Medicine, Faculty of Medicine, Aksaray University, Aksaray, Turkey; 7 Department of Medical Biochemistry, Faculty of Medicine, Aksaray University, Aksaray, Turkey

**Keywords:** Tramadol, Rutin, Lung toxicity, Oxidative stress, Apoptosis, Inflammation

## Abstract

Tramadol (TRM) is a commonly prescribed opioid analgesic; however, accumulating evidence suggests that it may exert toxic effects on vital organs, including the lungs. This study aimed to elucidate the mechanisms underlying TRM-induced lung injury and to investigate the potential protective role of rutin (RUT), a bioactive flavonoid with potent antioxidant and anti-inflammatory properties. In a rat model, lung tissues were analyzed using histopathological examination, biochemical assays for oxidative stress parameters, RT-qPCR for gene expression analysis [nuclear factor E2-related factor 2 (Nrf2), heme oxygenase-1 (HO-1), NAD(P)H:quinone acceptor oxidoreductase 1 (NQO1), nuclear factor kappa B (NF-κB), tumor necrosis factor alpha (TNF-α), inducible nitric oxide synthase (iNOS), Bax, Bcl-2, and Caspase-3], and immunohistochemical (IHC) evaluation of Beclin-1 and 3-nitrotyrosine (3-NT) expression. TRM administration caused severe pulmonary structural alterations, including alveolar collapse, interalveolar septal thickening, inflammatory infiltration, edema, and hemorrhage. These histopathological changes were associated with pronounced oxidative stress, as evidenced by suppressed superoxide dismutase (SOD), catalase (CAT), and glutathione peroxidase (GPx) activities, depletion of glutathione (GSH), increased lipid peroxidation, and disruption of the Nrf2/HO-1/NQO1 antioxidant signaling axis. Furthermore, TRM markedly activated endoplasmic reticulum (ER) stress responses (PERK and ATF-6), upregulated apoptotic markers (Bax and Caspase-3), downregulated Bcl-2 expression, and enhanced autophagy-related Beclin-1 immunoreactivity. In parallel, significant activation of inflammatory and nitrosative pathways was observed, characterized by elevated NF-κB, TNF-α, and iNOS expression and increased nitrotyrosine accumulation. In contrast, RUT treatment substantially ameliorated TRM-induced lung injury by restoring antioxidant capacity, suppressing ER stress-mediated apoptosis and autophagy, and attenuating inflammatory and nitrosative responses. Overall, these findings demonstrate that RUT confers significant protection against TRM-induced pulmonary toxicity through coordinated modulation of oxidative stress, ER stress, apoptosis, autophagy, and inflammation.

## Introduction

Tramadol (TRM) is a synthetic analgesic that exhibits weak agonist activity at the μ-opioid receptor and also inhibits the reuptake of norepinephrine and serotonin. Approved by the U.S. Food and Drug Administration (FDA) in 1995 for the treatment of moderate to severe pain, TRM exerts its analgesic effects through both opioid and monoaminergic pathways due to this dual mechanism of action (Hassamal et al. 2018). Recent studies have demonstrated that the analgesic effects of TRM are mediated not only through monoaminergic and opioid pathways but also via pain mediators such as transient receptor potential vanilloid 1 (TRPV1) channels, α2-adrenergic receptors, glutamate receptors, prostaglandin E2, voltage-gated sodium channels, proinflammatory cytokines, and adenosine receptors (Minami et al. 2015; Barakat 2019). Considering the multifaceted pharmacological properties of TRM, it is widely used in the alleviation of pain conditions with different etiologies, including low back pain, osteoarthritis, cancer-related pain, neuropathic pain, rheumatoid arthritis, labor, postoperative pain, and fibromyalgia (Subedi et al. 2019; Zeng et al. 2019). The ability of TRM to exert effects at both central and peripheral levels enhances its efficacy in pain syndromes associated with different pathophysiological mechanisms and provides a broad area of clinical use. Owing to these characteristics, TRM is considered an alternative or complementary option, particularly in patients requiring long-term analgesic treatment. Despite its widespread clinical use, TRM has also been reported to be associated with various adverse effects (April et al. 2019). The most commonly observed adverse effects include dizziness, nausea, vomiting, lightheadedness, sweating, fatigue, drowsiness, constipation, dry mouth, and sedation, and these effects generally occur in a dose-dependent manner (Beakley et al. 2015; Subedi et al. 2019). Rarer but clinically significant adverse effects include disorders related to the gastrointestinal and respiratory depression, immunological, cardiovascular systems, endocrine effects, hallucinations, and seizures (Lassen et al. 2015; Barakat 2019; Nakhaee et al. 2021). Such adverse effects are more frequently observed particularly with long-term use, at high doses, or when used concomitantly with other medications.

Inflammation is a complex biological response of the immune system to harmful stimuli such as pathogens, damaged cells, or toxic compounds, and it plays a critical role in the initiation and progression of many diseases. This process is characterized by the activation of immune cells and the release of proinflammatory mediators, including cytokines and inflammasomes such as the NLRP3 inflammasome, which regulate the production of key inflammatory cytokines (e.g., IL-1β and IL-18) (Li et al. 2025; Luo et al. 2025; Wang et al. 2025). Recent evidence suggests that dysregulation of inflammation and inflammasome-related pathways contributes significantly to tissue injury and disease pathogenesis, while modulation of oxidative stress and inflammatory signaling pathways such as NF-κB and NLRP3 may provide therapeutic benefits (Li et al. 2025; Luo et al. 2025).

Rutin (RUT) is a glycoside formed by the glycosylation of the flavonol-class aglycone quercetin with a rhamnose-glucose disaccharide and is chemically defined as 3,3′,4′,5,7-pentahydroxyflavone-3-rhamnoglucoside (Caglayan et al. 2019a; Choi et al. 2021). RUT is a polyphenolic flavonoid that is naturally found in many plants, vegetables, and fruits, including grapes, peaches, lemons, oranges, strawberries, buckwheat seeds, and tea (Kandemir et al. 2020a; Choi et al. 2021). One of the most important characteristics of RUT is its strong antioxidant structure; in addition, it stands out with numerous properties such as anti-inflammatory, antimicrobial, anticancer, neuroprotective, hepatoprotective, nephroprotective, and diabetes management supporting effects (Ganeshpurkar and Saluja 2017; Caglayan et al. 2019b; Çelik et al. 2020; Küçükler et al. 2021). Owing to these multifaceted pharmacological properties, RUT attracts attention as a potential protective agent.

The aim of this study was to investigate the possible protective effects of RUT in reducing TRM-induced lung injury. Accordingly, antioxidant parameters were analyzed to evaluate oxidative stress, and the expression levels of genes associated with inflammation, apoptosis, and endoplasmic reticulum (ER) stress were examined using the RT-qPCR method. In addition, the obtained findings were supported by immunohistochemical and histopathological examinations.

## Materials and methods

### Drug and chemicals

TRM (Contramal® Ampul 100 mg, Abdi Ibrahim, Istanbul) was obtained from a local pharmacy. RUT (≥ 94%) and other chemicals were purchased from Sigma Chemical Co. (St. Louis, MO, USA). All chemicals and reagents were of the highest purity grade.

### Experimental animals and ethical approval

A total of 28 Wistar albino rats weighing 220–250 g and aged 10–12 weeks were used in the experiment. The rats were obtained from Necmettin Erbakan University KONUDAM Experimental Medicine Application and Research Center. The animals were housed in cages in a controlled room maintained at a constant temperature of 24–25 °C with a 12 h light/12 h dark cycle (07:00–19:00 light; 19:00–07:00 dark). They were provided with unlimited access to water and standard laboratory chow. The rats were allowed to acclimatize to the environment for one week before the experiments were initiated. All experiments were conducted in accordance with the European Directive 2010/63/EU on the protection of animals used for scientific purposes. In addition, all procedures related to the animals used in this study were performed in accordance with Animal Research: Reporting of In Vivo Experiments (ARRIVE) guidelines. The animals used in the experiment were provided by Experimental Medicine Application and Research Center, Necmettin Erbakan University (Konya, Turkey) (Ethics Committee Approval No: 2025–058).

### Experimental design and doses applied to the study groups

Within the experimental protocol, TRM was administered to the experimental animals via the intraperitoneal (i.p.) route, while RUT was administered orally. Throughout the experimental period, the general health status and behavioral changes of the animals were monitored daily. Detailed information regarding the distribution of the experimental groups and the administration methods is presented below.

- Control Group: Saline was administered once daily for 14 days via oral and intraperitoneal (i.p.) routes.
- RUT Group: RUT was administered orally at a dose of 100 mg/kg once daily for 14 days (Kandemir et al. 2022).
- TRM Group: TRM was administered intraperitoneally at a dose of 50 mg/kg for 14 days (Karaca et al. 2025b).
- TRM + RUT Group: TRM was administered intraperitoneally at a dose of 50 mg/kg and RUT was administered orally at a dose of 100 mg/kg once daily for 14 days.

Twenty-four hours after the end of the experimental period, in accordance with ethical principles, the animals were euthanized under mild sevoflurane anesthesia and lung tissue samples were collected. A portion of the tissue samples obtained for the evaluation of antioxidant parameters and gene expression analyses (RT-qPCR) were stored at −80 °C until analysis. The remaining lung tissues were preserved in 10% formaldehyde for histopathological and immunohistochemical analysis.

### Biochemical analysis performed within the scope of the study

Within the scope of the biochemical evaluations, parameters related to lipid peroxidation and the antioxidant defense system were analyzed. For this purpose, malondialdehyde (MDA), catalase (CAT), superoxide dismutase (SOD), glutathione peroxidase (GPx), and glutathione (GSH) levels were determined in lung tissue. Prior to analysis, homogenates obtained from tissue samples were prepared as described in detail in our previous studies (Kandemir et al. 2020b; Erzincan et al. 2025). GPx activity was determined based on the method described by Lawrence and Burk (1976), SOD activity according to the method of Sun et al. (1988), GSH levels using the method of Sedlak and Lindsay (1968), CAT activity according to the Aebi (1984), and MDA levels in accordance with the protocols reported by Placer et al. (1966). Total protein content in lung homogenates was measured using the Lowry et al. (1951).

### Real-time PCR (RT-PCR)

At the end of the experimental process, the relative mRNA expression levels of the target gene regions specified in Table 1 in lung tissues obtained following TRM and RUT treatments were evaluated by quantitative real-time PCR (qRT-PCR) analysis. Total RNA isolation from lung tissues was performed using QIAzol Lysis Reagent (Qiagen, 79,306) in accordance with the manufacturer’s protocol. For complementary DNA (cDNA) synthesis from the obtained RNA samples, the OneScript Plus cDNA Synthesis Kit (ABM, G236, Richmond, Canada) was used. The synthesized cDNAs were combined with specific primer pairs and BlasTaq™ 2X qPCR MasterMix (ABM, G891, Richmond, Canada) to prepare the amplification reactions. qRT-PCR procedures were carried out on a Rotor-Gene Q (Qiagen) device under the thermal cycling conditions recommended by the manufacturer. The data obtained after amplification were normalized to β-actin, which was used as the reference gene, and relative gene expression changes were calculated based on the 2^−ΔΔCT^ method (Livak and Schmittgen 2001).

**Table 1:** RT-qPCR primer sequences used in this study

Gene	Sequences (5′−3′)	Length (bp)	Accession no
Nrf2	F: TTTGTAGATGACCATGAGTCGC R: TCCTGCCAAACTTGCTCCAT	161	NM_031789.2
HO-1	F: ATGTCCCAGGATTTGTCCGA R: ATGGTACAAGGAGGCCATCA	144	NM_012580.2
NQO1	F: CTGGCCAATTCAGAGTGGCA R: GATCTGGTTGTCGGCTGGAA	304	NM_017000.3
NF-κB	F: AGTCCCGCCCCTTCTAAAAC R: CAATGGCCTCTGTGTAGCCC	106	NM_001276711.1
TNF-α	F: CTCGAGTGACAAGCCCGTAG R: ATCTGCTGGTACCACCAGTT	139	NM_012675.3
iNOS	F: AGATCAATGCAGCTGTGCTC R: GGCTCGATCTGGTAGTAGTAGA	235	NM_012611.3
PERK	F: GATGCCGAGAATCATGGGAA R: AGATTCGAGAAGGGACTCCA	198	NM_031599.2
ATF-6	F: TCAACTCAGCACGTTCCTGA R: GACCAGTGACAGGCTTCTCT	130	NM_001107196.1
Caspase-3	F: ACTGGAATGTCAGCTCGCAA R: GCAGTAGTCGCCTCTGAAGA	270	NM_012922.2
Bax	F: TTTCATCCAGGATCGAGCAG R: AATCATCCTCTGCAGCTCCA	154	NM_017059.2
Bcl-2	F: GACTTTGCAGAGATGTCCAG R: TCAGGTACTCAGTCATCCAC	214	NM_016993.2
β-Actin	F: CAGCCTTCCTTCTTGGGTATG R: AGCTCAGTAACAGTCCGCCT	360	NM_031144.3

### Histological method

Following dissection, the lung tissue samples were fixed in 10% neutral buffered formalin solution for 24 h. After completion of fixation, the tissues were dehydrated through a graded ethanol series of 70%, 80%, 90%, 96%, and 100% to remove water. After dehydration, the samples were cleared in xylene and embedded in molten paraffin at 60 °C to obtain paraffin blocks. Sections of 5 µm thickness were cut from the paraffin blocks using a microtome and mounted on glass slides. For routine histological examination, the sections were stained with Hematoxylin–Eosin (H&E). After staining, the sections were dehydrated through a graded ethanol series (70–100%). The slides were cleared in xylene and mounted with entellan. The prepared slides were examined under a light microscope (Olympus CX43, Olympus Inc., Tokyo, Japan), and microscopic images were recorded using an EP50 digital camera system (Olympus Inc., Tokyo, Japan). The specimens were evaluated at a magnification of × 200, with a scale bar of 50 µm. A blinded scoring system was applied on H&E-stained sections based on the evaluation of lung edema, mononuclear cell infiltration, and hemorrhage. Histological changes were scored as follows: 0, 1, 2, 3, and 4, corresponding respectively to 0%, < 25%, 26–50%, 51–75%, and ≥ 76% injured/damaged lung tissue, and were graded on a scale of 0–4.

### Immunohistochemistry procedure

Paraffin sections of 3-µm thickness obtained from lung tissue samples used in the study were first deparaffinized with xylene and then rehydrated through decreasing concentrations of ethanol series. To unmask antigenic sites in the sections prepared for staining, antigen retrieval was performed by heat treatment in citrate buffer. To block endogenous peroxidase activity, the sections were incubated in 3% hydrogen peroxide solution for 10 min. Subsequently, the sections were washed with phosphate-buffered saline (PBS) and treated with a protein block solution for 10 min to prevent nonspecific binding. Following the blocking step, Beclin-1 (sc-48341, Santa Cruz Biotechnology) and nitrotyrosine (3-NT, sc-32757, Santa Cruz Biotechnology) primers diluted 1:100 in PBS were applied to the sections, and the samples were incubated overnight at + 4 °C. On the following day, the sections were washed three times with PBS for 5 min each, and then secondary antibody and streptavidin–biotin complex were applied sequentially. After each step, brief washing with PBS was performed. To visualize the immunoreaction, DAB (3,3′-diaminobenzidine) chromogen was applied to the sections, and the reaction was terminated by monitoring the development of a brown color. For contrast, the sections were counterstained with Harris hematoxylin for 5 min. Subsequently, the sections were dehydrated through 70%, 80%, and 96% ethanol, absolute alcohol, and xylene baths, respectively. After completion of all procedures, the slides were mounted with entellan and examined under a microscope. From each lung specimen in each rat group, ten randomly selected non-overlapping fields were examined under an Olympus CX43 light microscope (Olympus Inc., Tokyo, Japan) at 400 × magnification. The following grading system was used to calculate the percentage density of positive cells: staining intensity and distribution were graded as 0: negative staining; 1: mild immunoreactivity; 2: moderate immunoreactivity; and 3: widespread (strong) severe immunoreactivity. For scoring, the criteria of < 10% positive cells (1), 10–50% positive cells (2), and > 50% positive cells (3) were used. Immunoreactive cells and cell counts were estimated using image analysis software (ImageJ, version 1.46a, NIH, Bethesda, MD, USA).

### Statistical analysis

Statistical data obtained from the biochemical analyses in this study were evaluated using SPSS 20.0 software. Graphical presentations and additional statistical calculations were performed using GraphPad Prism 8 software. Differences between groups were analyzed using one-way analysis of variance (one-way ANOVA); when statistically significant results were obtained, pairwise comparisons between groups were performed using the Tukey post hoc test. A *p* value < 0.05 was considered statistically significant, and the data were presented as mean ± standard deviation (SD). Quantitative analyses of histopathological and immunohistochemical staining were performed using ImageJ software (version 1.46a; National Institutes of Health, Bethesda, MD, USA), and the results were expressed as mean ± SD.

## Results

### Effects of RUT on TRM-induced oxidative stress and lipid peroxidation in lung tissue

**Fig. 1:**
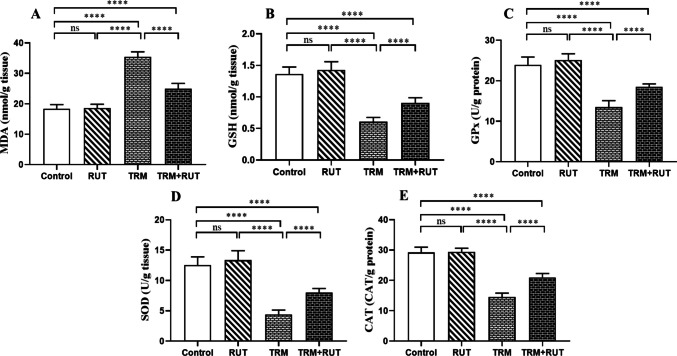
Effects of TRM and RUT treatments on (**A**) MDA and (**B**) GSH levels, as well as on the activities of the antioxidant enzymes (**C**) GPx, (**D**) SOD, and (**E**) CAT in lung tissue. Data are presented as mean ± SD. Statistical significance among all experimental groups is indicated by asterisks (****p < 0.0001), while ns denotes not significant differences

Analyses performed in lung tissue revealed that TRM exposure significantly impaired the oxidant-antioxidant balance (Fig. 1A–E). A marked reduction in CAT, SOD, and GPx activities, along with decreased GSH levels, was observed in all TRM-treated groups, whereas MDA levels were significantly elevated (*p* < 0.0001). These findings indicate enhanced lipid peroxidation and weakened antioxidant defense in response to TRM administration. In contrast, co-treatment of RUT notably attenuated TRM-induced oxidative damage. Compared with the TRM-only group, the TRM + RUT group exhibited significant increases in CAT, SOD, and GPx enzyme activities and GSH levels, accompanied by a pronounced decrease in MDA concentrations (*p* < 0.05).

**Fig. 2:**
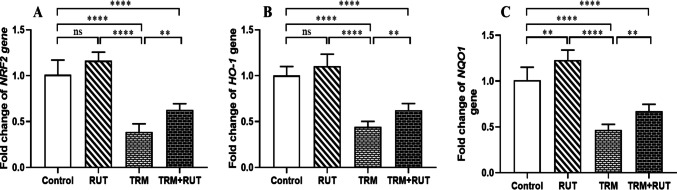
Effects of TRM and RUT treatments on lung tissue (**A**) NRF2, (**B**) HO-1, and (**C**) NQO1 expression levels. Data are presented as mean ± SD and expressed as fold changes relative to the control group. Statistical differences among experimental groups are indicated by asterisks (****p < 0.0001, **p < 0.01), whereas ns denotes non-significant differences

Consistent with the biochemical alterations, gene expression analyses demonstrated a significant downregulation of Nrf-2, HO-1, and NQO1 in lung tissue following TRM treatment (Fig. 2A–C). Notably, co-administration with RUT resulted in a significant upregulation of these antioxidant-related genes compared to the TRM group, suggesting activation of the Nrf-2 dependent antioxidant defense pathway.

### The effect of RUT on TRM-induced inflammation genes in lung tissue

**Fig. 3:**
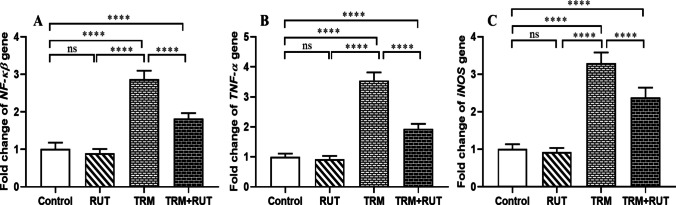
Lung tissue analyses demonstrated the effects of TRM and RUT treatments on the expression levels of (**A**) NF-κB, (**B**) TNF-α, and (**C**) iNOS. Results are presented as mean ± SD and are expressed as fold changes relative to the control group. Statistical significance among experimental groups is indicated by asterisks (****p < 0.0001), while ns represents non-significant differences

In this study, key signaling pathways associated with inflammatory responses were examined using complementary analytical approaches. The mRNA expression levels of NF-κB, TNF-α, and iNOS were assessed by RT-qPCR analysis. TRM exposure resulted in a marked upregulation of NF-κB, TNF-α, and iNOS transcripts (Fig. 3A–C), indicating activation of inflammatory signaling and increased cellular stress. In contrast, co-treatment with RUT significantly attenuated the TRM-induced transcriptional upregulation of these genes, suggesting that RUT effectively counteracts TRM-mediated inflammatory and stress-related molecular alterations.

### The effect of RUT on TRM-induced apoptotic genes in lung tissue

**Fig. 4:**
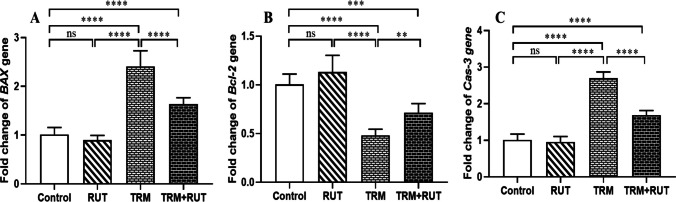
Lung tissue analyses demonstrated the effects of TRM and RUT treatments on the expression levels of (**A**) Bax, (**B**) Bcl-2, and (**C**) Cas-3. Results are presented as mean ± SD and are expressed as fold changes relative to the control group. Statistical significance among experimental groups is indicated by asterisks (****p < 0.0001, ***p < 0.001, **p < 0.01), while ns represents non-significant differences

The mRNA expression levels of Bax, Bcl-2, and Cas-3 were quantified by RT-qPCR analysis. Compared to the control group, TRM exposure significantly increased Bax and Cas-3 transcript levels (Fig. 4A–C), while Bcl-2 expression was markedly decreased, indicating activation of pro-apoptotic signaling pathways. Notably, when compared to the TRM group, the TRM + RUT group showed decreased Bax and Cas-3 expression levels and increased Bcl-2 expression, suggesting that RUT attenuates TRM-induced apoptosis by shifting the balance toward anti-apoptotic signaling.

### The effect of RUT on TRM-induced ER stress genes in lung tissue

**Fig. 5:**
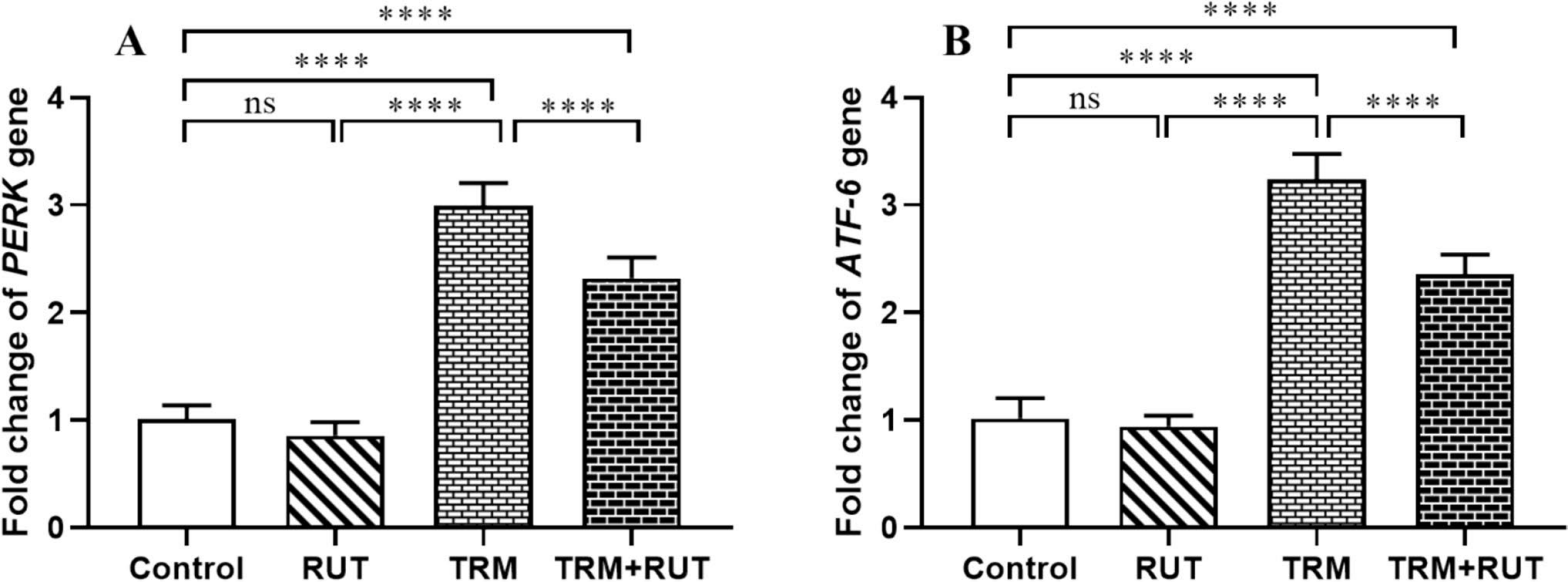
Lung tissue analyses demonstrated the effects of TRM and RUT treatments on the expression levels of (**A**) PERK and (**B**) ATF-6. Results are presented as mean ± SD and are expressed as fold changes relative to the control group. Statistical significance among experimental groups is indicated by asterisks (****p < 0.0001), while ns represents non-significant differences

To evaluate endoplasmic reticulum (ER) stress related responses, the mRNA expression levels of PERK and ATF-6 were analyzed. TRM treatment resulted in a significant upregulation of both PERK and ATF-6 expression levels, indicating activation of ER stress associated signaling pathways (Fig. 5A–B). In contrast, co-administration with RUT markedly attenuated these TRM-induced increases, as evidenced by reduced PERK and ATF-6 expression in the TRM + RUT group compared with the TRM-only group. These findings suggest that RUT exerts a protective effect by alleviating TRM-induced ER stress responses.

### Histological findings in lung tissue

**Fig. 6:**
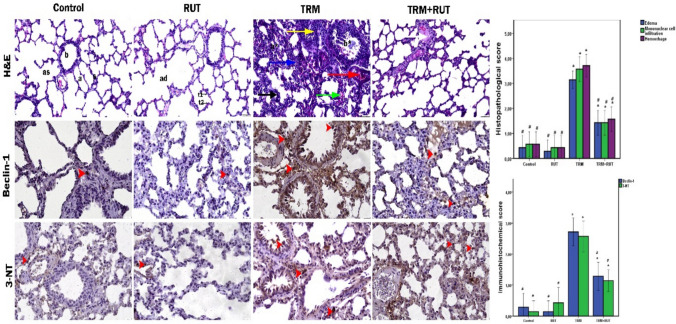
Photomicrographs of H&E and IHC-stained sections of lung tissues from control, RUT, and TRM-treated rats. Lung tissue from the control and RUT groups exhibits a typical pulmonary histo-architecture characterized by clear alveoli (**a**) lined with type I (t1) and type II (t2) pneumocytes in the bronchioalveolar unit parenchyma, well-defined alveolar sacs (as) and ducts (ad) formed by the alveoli, smoothly contoured bronchioles (**b**), and thin interalveolar septa (s). In the lungs of TRM-treated rats, dilated and distorted bronchioles (b*), collapsed alveoli (a*), thickening of the interalveolar septa (blue arrow), and intense mononuclear inflammatory cell infiltration in the peribronchial and perivascular areas (yellow arrow) are observed. In addition, dilation of congested blood vessels (red arrow), alveolar hemorrhage (green arrow), and an increased number of hyperchromatic nuclei in alveolar wall cells (black arrow) are noteworthy. In the lung tissues of the TRM + RUT group treated with rutin, mild edema (white arrow), the presence of thin interalveolar septa, and regular alveolar structures indicate that the normal histological architecture of the lung tissue has been restored. Furthermore, semi-quantitative histopathological scoring analysis revealed significant differences among the groups in the severity of pathological changes such as lung edema, inflammation, and hemorrhage. Beclin-1 immunoexpression: In the control and RUT groups, weak or limited cytoplasmic Beclin-1 positivity (arrowheads) is observed in alveolar epithelial and bronchiolar cells. In the TRM-treated group, marked cytoplasmic Beclin-1 staining and intense immunoreactivity (arrowheads) are evident in alveolar epithelial cells, bronchiolar epithelial cells, and the interstitial area. In the TRM + RUT group showing recovery after rutin treatment, Beclin-1 positivity is reduced (arrowheads), and expression is partially restored toward normal. Nitrotyrosine (3-NT) immunoexpression: While low-level or weak nuclear 3-NT staining is observed in the control and RUT groups (arrowheads), pronounced nuclear 3-NT immunoreactivity (arrowheads) is detected in alveolar and bronchiolar epithelial and endothelial cells in the TRM-treated group. In the TRM + RUT group, a significant reduction in 3-NT expression (arrowheads) is observed. Scale bar = 50 µm for H&E staining, 20 µm for IHC

The H&E staining results of lung tissue are presented in Fig. 6. In the lung tissues of the control and RUT groups, the normal histological structure was preserved; the alveolar spaces were regular and the alveolar walls appeared thin. No epithelial desquamation or debris was observed in the terminal and respiratory bronchioles. In contrast, marked histopathological alterations were detected in the micrographs of the TRM-treated group. A pronounced thickening of the alveolar septa was observed, and in some regions the alveolar spaces were narrowed and partially collapsed. Mononuclear cell infiltration, vascular congestion, interstitial hemorrhage, and edema were present in the interstitial area. In certain regions of the bronchiolar epithelium, epithelial cell shedding, an increased number of hyperchromatic nuclei, degenerative changes, and vacuole formation in epithelial cells were observed. In addition, mild perivascular inflammatory cell accumulation and dilation of capillary structures were detected in some samples. In the lung sections of the TRM + RUT group, overall tissue integrity was generally preserved and the alveolar structure was largely close to normal. The alveoli appeared clear and open in many areas, and no marked cellular debris or exudate accumulation was observed within the air spaces. Mild thickening of the interalveolar septa was noted, which was focal in some regions. The epithelial layer of the bronchiolar structures was preserved, with cells showing regular morphological alignment and patent lumens. Although mild congestion and edema were observed in capillary structures, widespread vascular dilatation was not detected. Furthermore, in the semi-quantitative evaluation, edema, inflammation, and hemorrhage scores in the TRM group were found to be significantly higher than those in the control group (*p* < 0.05). In the treatment (TRM + RUT) group, these scores were markedly reduced compared to the TRM group, and the histological structure was observed to approach normal.

### Immunohistochemical findings in lung tissue

The immunohistochemical staining results of lung tissue are presented in Fig. 6. In the lung tissues of the control and RUT groups, Beclin-1 immunostaining was very weak, showing only mild positivity in the cytoplasm of some alveolar and bronchiolar epithelial cells. No other marked immunoreactivity was observed. In contrast, a pronounced increase in Beclin-1 expression was observed in the TRM group. Intense cytoplasmic positivity was particularly evident in alveolar epithelial cells, bronchiolar epithelium, vascular endothelial cells, and some macrophages. The staining showed a widespread distribution along the alveolar walls, and strong immunoreactivity was observed in alveolar septal cells in some samples. In the TRM + RUT-treated group, Beclin-1 staining was milder and more limited, with positive cells confined to focal areas. Overall, immunoreactivity in this group was observed at a mild positivity level only in a subset of alveolar epithelial cells.

When 3-NT was evaluated, immunoreactivity in the lung sections of the control and RUT groups was quite weak and was observed only as mild nuclear staining in a few alveolar and bronchiolar epithelial cells. In the TRM-treated group, however, a significant increase in 3-NT immunoexpression was noted. Intense brown nuclear positivity was observed particularly in the nuclei of alveolar and bronchiolar epithelial cells, vascular walls, and some interstitial cells. In the group treated with the TRM + RUT drug combination, 3-NT positivity was markedly reduced. In this group, staining was generally confined to limited areas and predominantly exhibited weak positivity. Semi-quantitative scores for both Beclin-1 and 3-NT were significantly increased in the TRM group compared with the control group (*p* < 0.05). In the TRM + RUT group, the scores of both parameters were significantly decreased compared to the TRM group (*p* < 0.05).

## Discussion

Tramadol is an analgesic widely used on a global scale, and its mechanism of action is well established (Manouchehri et al. 2023). Although it has fewer gastrointestinal and renal adverse effects compared to NSAID analgesics, which provides an advantage in clinical use, its effects that are not clearly defined mechanistically and the reported serious adverse effects render TRM use controversial (April et al. 2019; Manouchehri et al. 2023; Seidmohammadi et al. 2025). In particular, the literature reports that TRM can induce toxic effects in vital organs such as the lungs, heart, and brain even at low therapeutic doses (Barbosa et al. 2021). In line with these literature data, an experimental model was established in the present study to demonstrate TRM-induced lung toxicity, and the potential preventive effects of RUT were evaluated in this model.

Oxidative stress is a well-established contributor to pulmonary injury; this study specifically focuses on the modulation of oxidative balance by TRM and RUT. Cells are equipped with endogenous antioxidant systems, including SOD, CAT, GPx, and GSH, which are critical for maintaining redox homeostasis (Bayav et al. 2024; Kandemir and Küçükler 2024; Caglayan et al. 2025). Similarly, the Nrf-2/HO-1/NQO1 signaling axis is widely recognized as a central regulator of cellular antioxidant responses and has been extensively characterized in previous studies (Ross and Siegel 2018; Consoli et al. 2021). Within this established framework, our findings provide evidence that TRM disrupts redox homeostasis at both molecular and biochemical levels. Specifically, TRM exposure significantly downregulated Nrf-2, HO-1, and NQO1 gene expression, accompanied by decreased SOD, CAT, and GPx activities and reduced GSH levels, alongside increased MDA levels, indicating enhanced lipid peroxidation. In contrast, RUT administration effectively mitigated these alterations, as demonstrated by the upregulation of antioxidant gene expression, restoration of enzymatic antioxidant activities, and suppression of lipid peroxidation. Importantly, these results emphasize the regulatory effect of RUT on TRM-induced oxidative imbalance rather than reiterating the established antioxidant pathways themselves. Consistent with our observations, previous studies have reported the protective role of RUT against oxidative lung damage (Bilgin et al. 2020; Ünver et al. 2021). Collectively, our data suggest that the protective efficacy of RUT is closely linked to its capacity to restore disrupted redox homeostasis in TRM-induced lung injury, thereby reinforcing its potential as a modulator of oxidative stress rather than merely confirming known mechanistic pathways.

In order to evaluate inflammatory and nitrosative stress responses, NF-κB, TNF-α, and iNOS gene expression levels were examined in lung tissue in this study, together with nitrotyrosine expression assessed by immunohistochemical methods. NF-κB is a key transcription factor that is activated under conditions of cellular stress and plays a central role in the regulation of inflammation, apoptosis, and oxidative stress responses (Lingappan 2018; Zhang et al. 2024). Increased NF-κB activation contributes to the amplification of the inflammatory response by transcriptionally inducing proinflammatory cytokines such as TNF-α and iNOS (Küçükler et al. 2021; Caglayan et al. 2022). In this study, the increases observed in NF-κB, TNF-α, and iNOS gene expression following TRM administration suggest that a pronounced inflammatory and nitrosative stress response was activated in lung tissue. The increase in nitrotyrosine expression, a biochemical indicator of this process, was immunohistochemically confirmed in our study and revealed that TRM markedly increased nitrosative damage in lung tissue. Nitrotyrosine accumulation is an important pathological marker that contributes to the impairment of protein function, disruption of cellular signal transduction, and deterioration of tissue integrity (Ahsan 2013). On the other hand, the significant suppression of NF-κB, TNF-α, and iNOS gene expression and the reduction of nitrotyrosine immunoexpression by RUT administration support the strong anti-inflammatory and anti-nitrosative effects of this flavonoid. This protective effect is thought to have occurred through inhibition of the NF-κB signaling pathway by RUT and limitation of reactive nitrogen species formation. When all these findings are evaluated together, it can be concluded that inflammatory and nitrosative stress mechanisms play an important role in TRM-induced lung injury, whereas rutin administration may alleviate tissue damage by suppressing these pathological processes.

ER stress is one of the fundamental cellular adaptive mechanisms that can be activated to maintain cellular homeostasis (Gül et al. 2025). ER stress, which can be triggered by various pathophysiological stimuli including oxidative stress, is generally defined as a stress response that arises as a result of the accumulation of unfolded or misfolded proteins within the ER lumen (Emre Kızıl et al. 2023; Varışlı et al. 2023). This stress response is initiated through sensor proteins such as PERK and ATF6 located on the ER membrane, leading to the activation of the unfolded protein response (UPR) in order to restore cellular balance. However, when ER stress persists for a prolonged period or becomes severe and adaptive mechanisms are insufficient, dysfunction of these sensor proteins may occur, causing the stress response to shift toward pro-apoptotic and/or pro-autophagic signaling pathways (Song et al. 2018; Kandemir et al. 2025). These processes are defined in the literature as type 1 (apoptosis) and type 2 (autophagy) programmed cell death mechanisms, respectively (Hajibabaie et al. 2023). Particularly under conditions where ER stress cannot be resolved, the pro-apoptotic transcription factor CHOP is activated and is known to play a central role in the apoptotic process. CHOP activation suppresses anti-apoptotic Bcl-2 expression via Bid and disrupts the Bax/Bcl-2 balance, thereby triggering apoptotic cell death through Caspase-3 activation via the mitochondrial pathway (Kandemir et al. 2025; Bayav et al. 2026). Although the molecular mechanisms of ER stress–related autophagic cell death are less well elucidated compared to apoptosis, Beclin-1 is suggested to play a critical regulatory role in this process (Song et al. 2018). Beclin-1 is a protein of central importance in the initiation of autophagy, phagophore nucleation, and autophagosome formation processes (Kang et al. 2011). In this context, the interaction between ER stress and autophagy is thought to be a determining factor in the regulation of cellular fate. It has been reported in the literature that TRM administration can trigger ER stress (Bülbül et al. 2026; Karaca et al. 2025a). In addition, studies have demonstrated that TRM induces apoptosis and autophagic cell death in various tissues (Barbosa et al. 2021; Bülbül et al. 2026; Karaca et al. 2025a, 2025b). In the present study, PERK and ATF6 gene expression levels were examined to evaluate the ER stress response in lung tissue; Bax, Bcl-2, and Caspase-3 gene expression levels were assessed to elucidate the apoptotic process; and Beclin-1 expression was investigated by immunohistochemical methods in terms of autophagic cell death. The obtained findings clearly demonstrated that TRM administration increased ER stress based on the evaluated parameters, thereby triggering apoptosis and autophagy and leading to marked damage in lung tissue. In contrast, RUT administration was observed to significantly reduce lung tissue damage by suppressing these molecular alterations.

The histopathological findings obtained indicate that TRM administration causes marked structural damage in rat lung tissue. Alveolar septal thickening accompanied by narrowing and partial collapse of the alveolar spaces suggests that the gas exchange surface is adversely affected. The accompanying interstitial mononuclear cell infiltration, vascular congestion, hemorrhage, and edema suggest the development of an active inflammatory response in lung tissue. Moreover, the observation of degenerative changes in the bronchiolar epithelium, epithelial cell shedding, and vacuolar formations, together with perivascular inflammation and capillary dilatation, supports the notion that TRM disrupts epithelial and vascular integrity, thereby exacerbating pulmonary injury. Following RUT administration, a marked reduction in TRM-induced lung damage was observed. Histological evaluation demonstrated better preservation of alveolar and bronchiolar structures, along with decreased inflammatory cell infiltration and interstitial edema. These findings indicate that RUT exerts a protective effect against TRM-induced pulmonary toxicity. Indeed, Shahid et al. reported that RUT exhibits significant protective properties in mouse lung tissue (Shahid et al. 2016); similarly, Verma et al. reported a protective effect of a RUT-containing compound against radiation-induced lung injury (Verma et al. 2017). The protective effect of RUT is thought to be associated with its strong antioxidant and anti-inflammatory properties, through which it suppresses oxidative and inflammatory stress responses.

## Conclusion

**Fig. 7:**
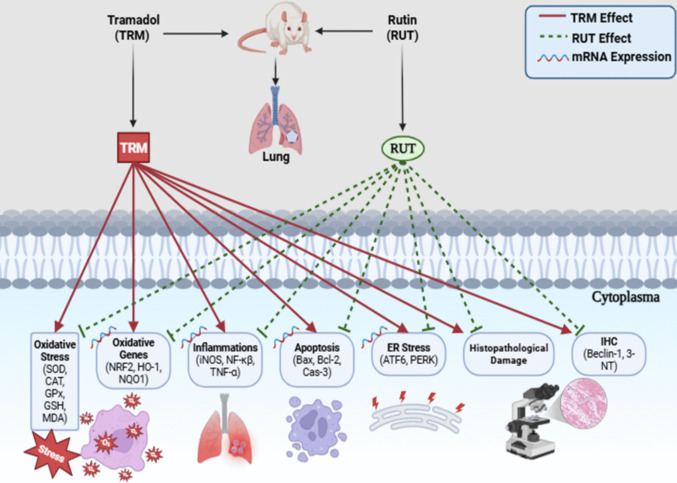
Mechanistic illustration showing the ameliorative effects of rutin (RUT) against tramadol (TRM)-induced lung injury through modulation of oxidative stress, inflammation, apoptosis, and endoplasmic reticulum stress in rats. Created with BioRender.com

In conclusion, the present study demonstrates that TRM induces severe lung injury characterized by pronounced structural damage, oxidative imbalance, endoplasmic reticulum stress, apoptosis, autophagy, and inflammatory and nitrosative responses. The concomitant disruption of the Nrf2/HO-1/NQO1 antioxidant axis and activation of ER stress–related apoptotic and autophagic pathways appear to play a central role in the pathogenesis of TRM-induced pulmonary toxicity. Importantly, RUT treatment markedly ameliorated these detrimental effects by restoring antioxidant defense mechanisms, suppressing ER stress–mediated cell death signaling, and attenuating inflammatory and nitrosative stress responses. These findings highlight RUT as a promising protective agent against TRM-induced lung injury and provide mechanistic insight into its multifaceted cytoprotective actions. This mechanistic framework is summarized in Fig. 7, which illustrates the interconnected regulation of oxidative stress, inflammation, apoptosis, and ER stress that underlies the ameliorative effect of RUT. Collectively, this study contributes to a better understanding of the molecular events underlying TRM-related pulmonary toxicity and suggests that RUT may represent a potential adjunctive therapeutic strategy to mitigate opioid-induced lung damage.

### Limitations

Despite the comprehensive evaluation of oxidative stress, ER stress, apoptosis, autophagy, and inflammatory pathways, this study has several limitations that should be acknowledged. First, the mechanistic insights were derived primarily from gene expression analyses and immunohistochemical assessments; therefore, confirmation at the protein and functional levels using techniques such as Western blotting or activity-based assays would further strengthen the conclusions. Second, only a limited number of ER stress, apoptotic, and autophagic markers were examined, and additional upstream and downstream regulators could provide a more detailed understanding of these pathways. Finally, the study was conducted in an experimental rat model, and extrapolation of the findings to clinical settings should be approached with caution. Future studies incorporating different doses, time points, and translational models are warranted to validate and extend these findings.

## Data Availability

All source data for this work (or generated in this study) are available upon reasonable request.
